# Enhanced coverage by integrating site interdependencies in capacitated EMS location models

**DOI:** 10.1007/s10729-021-09562-4

**Published:** 2021-07-13

**Authors:** Matthias Grot, Tristan Becker, Pia Mareike Steenweg, Brigitte Werners

**Affiliations:** 1 Institute of Management ifu, Ruhr University Bochum, Universitätsstraße 150, 44801 Bochum, Germany; 2 Chair of Operations Management, RWTH Aachen University, Kackertstr. 7, 52072 Aachen, Germany

**Keywords:** Emergency medical services, Facility location, Site interdependency, Busy fraction, Operations Research

## Abstract

In order to allocate limited resources in emergency medical services (EMS) networks, mathematical models are used to select sites and their capacities. Many existing standard models are based on simplifying assumptions, including site independency and a similar system-wide busyness of ambulances. In practice, when a site is busy, a call is forwarded to another site. Thus, the busyness of each site depends not only on the rate of calls in the surrounding area, but also on interactions with other facilities. If the demand varies across the urban area, assuming an average system-wide server busy fraction may lead to an overestimation of the actual coverage. We show that site interdependencies can be integrated into the well-known Maximum Expected Covering Location Problem (*MEXCLP*) by introducing an upper bound for the busyness of each site. We apply our new mathematical formulation to the case of a local EMS provider. To evaluate the solution quality, we use a discrete event simulation based on anonymized real-world call data. Results of our simulation-optimization approach indicate that the coverage can be improved in most cases by taking site interdependencies into account, leading to an improved ambulance allocation and a faster emergency care.

## Highlights

- We propose a new mixed-integer programming model for emergency medical services (EMS) systems incorporating site interdependencies.
- To evaluate EMS service quality, we develop a simulation model based on a real-life EMS system.
- Computational studies show increased service quality when modeling site interdependencies.
- Further, results from a case study reveal improved service quality for a real-world application.

## Introduction

In case of medical emergencies, patients require fast and qualified assistance. From a patients point of view, the response time of paramedics depends primarily on the location of the nearest ambulance. These locations are typically fixed on a strategic level and there may be several ambulances at each one. If an ambulance is available at the closest site, that site will answer an incoming emergency call. Otherwise, if all ambulances of that site are busy, the call is transferred to the second closest site in the system and so on. Therefore, the availability of a site has a great influence on the response time of emergency calls. By locating multiple ambulances at one site, the probability of the site being available increases.

The quality of an EMS system is typically measured using *coverage*, defined as the proportion of emergency calls that are reached within a given time threshold. Coverage targets vary across countries and regions [1]. In our study, we use the target to reach at least 90% of calls within 10 minutes, which is common for German urban areas. A high service quality is achieved when there is a high probability that an ambulance is available at the site closest to a call. Therefore, a given number of ambulances is to be allocated to sites so that the level of coverage is maximized.

We measure the time threshold from the time an incoming call occurs until the first ambulance arrives at the location of the emergency. In this context, we differentiate between pre-travel delay and travel time. We define the pre-travel delay as the time between the arrival of an incoming call and the departure of an ambulance. The travel time is the time an ambulance needs to reach the location of the emergency after departure from its site. Thus, an optimal distribution of ambulances over the urban area is important for a high quality EMS system.

Due to the practical relevance, there is a rich body of literature on EMS location problems. Standard models maximize, e.g., the coverage (Maximal Covering Location Problem (*MCLP*)) [2] or the expected coverage of emergencies using busy fractions (Maximum Expected Covering Location Problem (*MEXCLP*)) [3]. The busy fraction denotes the proportion of time a resource (an ambulance, a site, or the system) is in service and therefore not available for incoming emergency calls. Further research focuses on the availability of the system (Maximal Availability Location Problem (*MALP*)) [4]. These standard models make several simplifying assumptions on, e.g., server busyness, server independence, or location-independent service times [5]. These assumptions are used to deal with the challenges that arise from the probabilistic nature of EMS systems and to obtain tractable linear mathematical formulations. There are many extensions of these standard formulations that aim at representing the emergency demand and objectives of a city or area more realistically.

One central assumption that limits the adequate representation of reality and, thus, is often addressed by these extensions, is the average system-wide server busy fraction. Urban areas are typically characterized by a heterogeneous spatial distribution of emergency calls - the frequency of calls at the city center is higher than in the suburbs. Thus, the busy fraction is different for each ambulance throughout an urban area. If a uniform (i.e., a system-wide identical) busy fraction is assumed, given a similarly sized area of coverage for each site, the actual busy fraction of sites close to the city center tends to be higher, while the busy fraction of suburban sites is lower. This simplifying assumption of the *MEXCLP* tends to lead to an overestimation of the expected coverage at the city center and an underestimation in the suburbs. In practice, ambulances in the suburbs less frequently face emergency calls compared to ambulances in the city center. Thus, these less frequented ambulances often serve as a back-up for emergency events that occur in the city center, but need more time to reach the emergency. The result is an inefficient allocation of the limited resources of the EMS system due to the simplifying assumption of a system-wide busy fraction. To improve the standard models for the case of heterogeneous demand regions, a specified system-wide busy fraction must be adhered at every single site. This upper bound for capacity utilization limits the number of assigned emergency calls for each site depending on its number of ambulances. Whenever discussing ’capacity’ in the context of capacitated EMS location models, we follow this interpretation.

**Fig. 1:**
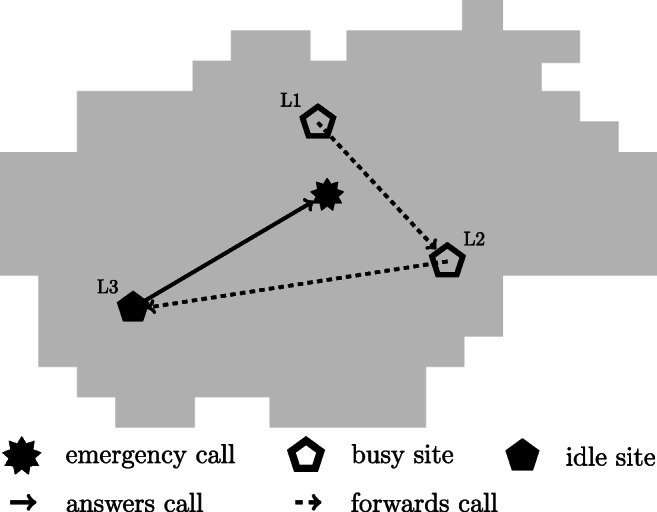
Site interdependencies in EMS networks

One main simplifying assumption in standard models is the site or ambulance independency, i.e., neglecting the interaction effects between sites or ambulances. However, if the closest site cannot answer an incoming emergency call, the call is transferred within the system with predetermined priorities (levels) as depicted in Fig. 1 and the assumption of independently operating ambulances may not be strictly true in reality [6]. In most cases, the priority of a site decreases with an increasing distance to the emergency location. Thus, an unavailable site is supported by other available sites and vice versa takes calls from other sites. These interdependencies are modeled in a formulation with a system-wide busy fraction as an upper bound for each specific ambulance by Ansari et al. [7] and Shariat-Mohaymany et al. [8]. The back-up assistance on lower priority levels has to be included in the calculation of the busy fraction. In contrast to [7, 8], we impose a system-wide upper bound on the busy fraction of each site in the strategic optimization model while ambulances in the system may have different busy fractions.

This leads to the following research question: How can we model site interdependencies in EMS location models and what is the impact on service quality? To answer this question, we propose a simulation-optimization approach with an innovative mixed-integer linear optimization model based on the *MEXCLP* accounting for a maximum busy fraction per site by the introduction of capacity constraints. In extensive computational experiments, we show that the consideration of site interdependencies leads to an improved model performance compared to existing standard models. The remainder of this paper is structured as follows: Section 2 gives a literature review of the basic EMS location models and the related chance constraint and capacitated approaches. The innovative mathematical formulation is presented in Section 3. In Section 4 extensive computational experiments evaluate our new model formulation before a detailed case study with anonymized real-world data is conducted in Section 5. Section 6 concludes the paper and gives an outlook on further research.

## Literature review

### Basic EMS location models

Mathematical optimization models are frequently applied to support decision-making in emergency medical services, examples related to staff planning or location problems are presented in [9, 10]. There are several literature reviews that provide a comprehensive overview of various aspects related to EMS. A review of covering models and optimization techniques for the planning of EMS facility locations is provided by Li et al. [11]. A recent and extensive overview of many important aspects for EMS systems is given by Aringhieri et al. [12]. They consider the entire chain of events from emergency call to recovery in the hospital. Ahmadi-Javid et al. [13] provide another comprehensive review with a focus on emergency and non-emergency healthcare facility location problems.

Most publications deal with stochastic EMS location problems. This work extends classical EMS location models to consider heterogeneous busyness over the urban area and model site interdependencies. Thus, our review focuses on ambulance location models and probabilistic extensions.

Since the early 1970s, several deterministic EMS location models have been proposed [2, 14–16]. Despite their shortcomings, deterministic models have nevertheless enjoyed great popularity in many applications, due to their relative simplicity of implementation. Particularly in cases with a low population density, few resources are distributed over a large geographical area and probabilistic effects are less important. However, there is a number of studies that extend deterministic models by probabilistic effects. The Maximum Expected Covering Location Problem (*MEXCLP*) by Daskin [3] and the Maximum Availability Location Problem (*MALP*) by ReVelle/Hogan [4] represent two seminal studies in this area. In addition to applications in the context of EMS, there are other applications of the *MEXCLP* as, e.g., locating fire and police stations, as well as large-scale emergency and disaster events [17, 18]. The *MEXCLP* and *MALP* extend the *MCLP* and include a busy fraction *q*, i.e., the fraction of time a server is busy with a call and therefore not available to answer an additional call. This is the probability that a call cannot be answered by the server.

The *MEXCLP* maximizes the fraction of demand that is covered within a certain time threshold considering server busyness. Integer variable *x*_*i*_ specifies the number of servers at site *i*. Binary variable *y*_*jk*_ denotes whether demand node *j* is covered by a number of *k* servers that are positioned at facilities *i* ∈ *N*_*j*_ that reach node *j* within the time threshold. The number of calls at demand node *j* is specified by *d*_*j*_ and a total number of *V* servers is available. The uniform server busy fraction *q* is obtained by assuming that the system-wide workload is evenly distributed among the *V* servers. The *MEXCLP* is then defined as follows [3]:

1 $$ \begin{array}{@{}rcl@{}} &\max \sum\limits_{j \in J} \sum\limits_{k \in K} (1 - q) q^{k-1} d_{j} y_{jk} & \end{array} $$

2 $$ \begin{array}{@{}rcl@{}} &\textrm{s.t.} \sum\limits_{k \in K} y_{jk} \leq \sum\limits_{i \in N_{j}} x_{i} & \forall j \in J \end{array} $$

3 $$ \begin{array}{@{}rcl@{}} & \sum\limits_{i \in I} x_{i} = V \end{array} $$

4 $$ \begin{array}{@{}rcl@{}} & x_{i} \in \mathbb{Z}^{\geq0}; y_{jk} \in \{0, 1\} & \forall i \in I, j \in J, k \in K \end{array} $$

The additional fraction of demand covered by adding server *k* is (1 − *q*)*q*^*k*− 1^. Objective (1) maximizes the fraction of demand covered within the specified distance, taking into account average server unavailability. Constraints (2) ensure that demand node *j* is only covered by *k* servers if a number of *k* servers are positioned at the facilities *i* ∈ *N*_*j*_. Constraint (3) ensures that a number of *V* servers is positioned at the available facilities. The *MEXCLP* requires several simplifying assumptions, in particular that all servers are uniformly busy and they are independent. The probability of server unavailability is modeled by parameter *q* and does not depend on the decision variables. In fact, the actual busy probability of each site $\tilde {q}^{i}$ depends on the number of calls in proximity to the location *i*. Furthermore, it depends on the location and capacity of other sites in the proximity because they influence the total number of calls that arrive, including back-up calls. Thus, the objective function of *MEXCLP* may over- or underestimate the actual coverage. There already exist some extensions of the *MEXCLP* on improving the accuracy of the estimation of expected coverage by relaxing some of the assumptions, i.e., server independency and location independent uniform server busy fractions (see e.g., [6, 19]). However, Saydam et al. [20] compare different MEXCLP extensions and conclude that none of the compared extensions is consistently superior in terms of a more accurate estimation of the expected coverage. We, too, develop an extension of *MEXCLP* but model coverage more precisely.

### Chance constraint approaches

For modeling uncertainties in the EMS context, chance constraint approaches based on the idea of the *MALP* formulation by ReVelle/Hogan [4] are widely used. These models have in common that a set of constraints is met with *α* reliability. Their chance constraint requires that at least one server is available in the system with a probability greater or equal than *α*. Note that the busy fraction is 1-*α*. Uncertainty in the EMS context mostly refers to answering emergency calls within a given time threshold while some ambulances may already be busy. Chance constraint approaches are widely used in the context of EMS (see e.g., [21–26]).

Following the idea of these chance constraint approaches, this paper presents a new type of linear capacity constraint that ensures a given busy fraction for each site to be kept. Similar to the chance constraints of ReVelle/Hogan [4] we state that each site has at least one server available with a given probability. Thus, there is an upper bound on the probability that a site cannot answer to an incoming call. Shariat-Mohaymany et al. [8] adopt this idea of reliability constraints in a similar manner. They propose a linear model that provides a pre-specified minimum reliability level for each demand node based on an upper probability limit for the busy fraction of each ambulance. Other approaches based on chance constraints are, e.g., [5, 27–29]. Our paper uses a similar reliability approach to Shariat-Mohaymany et al. [8] with the main differences that we consider ambulances to have different busy fractions and sites to operate interdependently.

### Capacitated extensions

There are many extensions of the original *MEXCLP* and *MALP* models in the EMS context. These extensions include time dependencies (see e.g., [30, 31]), ambulance relocations (see e.g., [10, 32]), probabilistic coverage (see e.g., [33–35]), or patient survival (see e.g., [36–38]).

Furthermore, some extensions comprise capacity constraints to which our approach belongs. These models allow for allocating additional demand to a site if and only if there is enough capacity, i.e., idle servers, available at that site. One of the first publications addressing this issue was published in 1991 by Pirkul/Schilling [39] based on the capacitated version of the *MCLP* model by Current/Storbeck [40]. They propose a backup based model that restricts the total service provided by each site to a specified service capacity. To solve the model, several simplifying assumptions are necessary, e.g., one server per site, one backup level, no interaction effects between servers as well as capacity limits. Araz et al. [41] extend this approach by a fuzzy multi-objective model that relaxes some of the restrictive assumptions, e.g., they allow for multiple servers per site. Recently, Raghavan et al. [42] have presented the Capacitated Mobile Facility Location Problem (*CMFLP*). In their approach, there is a limit on the number of clients that may be allocated to a server. In a similar manner, Shariat-Mohaymany et al. [8] propose an upper bound that limits the utilization of servers to a predefined maximal busy fraction. One central assumption of independently operating servers remains.

All these approaches neglect some of the system dynamics, e.g., server interdependencies, which could lead to errors in determining the level of coverage. Ansari et al. [7] propose a model that relaxes the assumption of independently operating ambulances. The Maximum Expected Covering Problem for District Design (*MECPDD*) is a stochastic model that accounts for travel time and server availability uncertainties and balances the busyness among the servers by introducing lower and upper capacity bounds on the servers. Thus, this model formulation also follows the idea of limiting server capacity by a maximum busy fraction. To solve the *MECPDD* an iterative algorithm is presented based on the hypercube queueing model by Larson [43]. In contrast, our approach limits site capacities, leading to a reduced model complexity, and can therefore be solved with a commercial solver, also taking interdependencies into account.

### Contribution

This paper proposes a new linear optimization model accounting for limited site capacities to ensure a maximum busy fraction. In our simulation-optimization approach, we use a discrete event simulation to evaluate the performance of the model solutions in order to identify the upper bound on the busy fraction that provides the highest coverage. The effect of forwarded calls on the busyness of a site is considered and thus site interdependencies are explicitly modeled under the assumption of statistical server independence. Furthermore, we propose two extensions of the *MEXCLP* with probabilistic coverage and capacity constraints. In this way, our computational experiments can separately quantify the advantage of modeling probabilistic coverage and capacity constraints. Thus, the contribution of the paper is fourfold:

1. Modeling of site interdependencies and capacity utilization,
2. Innovative mathematical formulation which imposes an upper bound on the site busy fraction,
3. Mixed-integer linear optimization model that is solved using commercial solvers,
4. Validation of the formulation in a large computational experiment and case study with anonymized real-world data, showing improved coverage.

We are not aware of any previous linear mathematical formulations that integrate site interdependencies by introducing a uniform capacity upper bound for each site.

In contrast to the idea of Shariat-Mohaymany et al. [8] that limit demand capacities for each server, we suggest an extension of the upper bound capacity utilization approach that integrates site interdependencies by limiting the demand capacities for each site. As opposed to the iterative hypercube approach by Ansari et al. [7], we use a level based approach to handle the non-linearities imposed by the probabilistic nature of site interdependencies. Thereby, we are able to solve the proposed model using commercial solvers. Further, our approach can be integrated into existing EMS models to provide a more realistic representation of the EMS system.

## Mathematical formulation

This section presents the proposed Capacitated Maximum Expected Covering Location Problem (*CMEXCLP*). Preliminary considerations focus on the capacitated extension of the *MEXCLP* taking into account site interdependencies. To avoid redundancies, the notation is adopted from the previous section, unless otherwise stated. A complete overview of the notation can be found in the Appendix.

### Modeling interdependencies

A realistic representation of an EMS system often leads to complex and non-linear mathematical formulations. To maintain a linear formulation, models assume values for the busy fraction that are based only on system-wide demand possibly resulting in inefficient ambulance allocations.

By introducing an upper bound for the busyness of each site, i.e., the probability that all servers located at a site are busy, this error can be limited. In contrast to limiting the busyness of each ambulance, limiting the busyness of each site allows for different busy fractions for individual ambulances. Furthermore, it is possible to explicitly model site interdependencies in the capacity constraints while maintaining a linear model. Additionally, the resulting linear model formulation is more compact compared to a server oriented approach as there are usually at least as many ambulances as sites in an EMS system. Let $\bar {t}$ be the average duration (in hours) of a call and let *d*_*j*_ denote the number of calls per day at demand node *j* ∈ *J*. Then, the average busy fraction of a server *q* can be derived as follows, taking into account that there are a number of *V* servers and 24 hours per day:

5 $$ \begin{array}{@{}rcl@{}} q = \frac{\bar{t}\cdot{\sum}_{j \in J} d_{j}}{24\cdot V} \end{array} $$

There is an inverse relationship of the busy fraction of a site and the number of servers *V*_*i*_ at a site *i* ∈ *I*. Furthermore, the site busy fraction increases with the allocated demand *f*_*ij*_ from demand node *j* ∈ *J*. To ensure that the site busy fraction does not exceed a value of *q*^*ub*^, the following inequalities can be deduced for each site *i* from equation (5):

6 $$ \begin{array}{@{}rcl@{}} \left( \frac{\bar{t}\cdot{\sum}_{j \in J} f_{ij}}{24\cdot V_{i}}\right)^{V_{i}} \leq q^{ub} \quad \forall i \in I \end{array} $$

The numerator on the left-hand side of inequalities (6) denotes the expected time consumed by answering calls during a day where the number of calls that are served is given by ${\sum }_{j \in J} f_{ij}$. The denominator captures the number of ambulance hours which are available over the course of each day. The maximum amount of demand that can be served while maintaining a busy fraction *q*^*ub*^ depends only on the number of servers *V*_*i*_. Thus, inequalities (6) can be rewritten as follows:

7 $$ \begin{array}{@{}rcl@{}} \bar{t}\cdot\sum\limits_{j \in J} f_{ij} \leq 24 \cdot V_{i} \sqrt[V_{i}]{q^{ub}} \quad \forall i \in I \end{array} $$

Note that the right-hand side of inequalities (7) states the maximum capacity of a site *i* ∈ *I* given a number of *V*_*i*_ servers and a maximum busy fraction of *q*^*ub*^. Given this upper bound for the busy fraction, it is possible to integrate site interdependencies.

The priority a site *i* has when an emergency occurs at demand node *j* is specified as the level *l* of coverage. A site that covers a demand node with *l* = 1 is requested first when an emergency occurs. If that site is busy, the site with *l* = 2 responds. Let *L* denote the set of possible levels *l* of coverage. Binary variable *y*_*ijl*_ = 1 if site *i* provides coverage for demand node *j* on level *l*, and *y*_*ijl*_ = 0 otherwise. Moreover, binary variable *x*_*ik*_ = 1 if site *i* has server *k*, and *x*_*ik*_ = 0 otherwise. In addition, let *c*_*ij*_ denote the average time required to serve an emergency at demand node *j* when answered by site *i*. Note that *c*_*ij*_ substitutes $\bar {t}$ as level based site specific travel times are considered. This relaxes the assumption of a site independent average call duration as travel times might be different depending on the location of the responding site. Finally, we propose the following capacity constraints considering site interdependencies to ensure a maximum busy fraction of *q*^*ub*^:

8 $$ \begin{array}{@{}rcl@{}} &&\sum\limits_{j \in J} \sum\limits_{l \in L} {q^{ub}}^{(l-1)} (1 - q^{ub}) c_{ij} d_{j} y_{ijl} \leq \\ && \sum\limits_{k \in K} (24 \cdot k \sqrt[k]{q^{ub}} - 24 \cdot (k-1) \sqrt[(k-1)]{q^{ub}}) x_{ik}   \forall i \in I \end{array} $$

The left-hand side of constraints (8) captures the busyness of site *i*. Given a first level assignment, a site has to serve a fraction of 1 − *q*^*ub*^ of the demand (*d*_*j*_). On each successive level, the probability that a call is forwarded is the product of the busy fractions of all preceding facilities and the probability that the site is available. The maximum amount of demand that is forwarded from the preceding levels is thus defined by *q*^*ub*^^(*l*− 1)^(1 − *q*^*ub*^). Thus, the maximum busy fraction of a site is *q*^*ub*^ if constraint (8) is met with equality. If a lower amount of demand is assigned, the busy fraction is strictly lower than *q*^*ub*^. The right-hand side specifies the capacity of site *i* in terms of the maximum amount of demand that can be served given *k* servers while a busy fraction of *q*^*ub*^ is maintained. These preconsiderations lead to the mathematical formulation of the new capacitated *MEXCLP* (*CMEXCLP*).

### Capacitated Maximum Expected Covering Location Problem (*CMEXCLP*)

The *CMEXCLP* extends the *MEXCLP* by capacity constraints to ensure a maximum busy fraction and to account for site interdependencies as well as for probabilistic coverage.

9 $$ \begin{array}{@{}rcl@{}} &{}\max\quad & \sum\limits_{i \in I} \sum\limits_{j \in J} \sum\limits_{l \in L} (1 - q^{ub}) {q^{ub}}^{(l-1)} d_{j} p_{ij} y_{ijl} \end{array} $$

10 $$ \begin{array}{@{}rcl@{}} &s. t. & \sum\limits_{i \in I} \sum\limits_{k \in K} x_{ik} = V \end{array} $$

11 $$ \begin{array}{@{}rcl@{}} &&x_{ik} \leq x_{i(k-1)} \quad \forall i \in I, k \in K\ |\ k > 1 \end{array} $$

12 $$ \begin{array}{@{}rcl@{}} && \sum\limits_{i \in I} y_{ijl} = 1 \quad \forall j \in J, l \in L \end{array} $$

13 $$ \begin{array}{@{}rcl@{}} &&y_{ijl} \leq x_{i1} \quad \forall i \in I, j \in J, l \in L \end{array} $$

14 $$ \begin{array}{@{}rcl@{}} && \sum\limits_{l \in L} y_{ijl} \leq 1 \quad \forall i \in I, j \in J \end{array} $$

15 $$ \begin{array}{@{}rcl@{}} &&\sum\limits_{j \in J} \sum\limits_{l \in L} {q^{ub}}^{(l-1)} (1 - q^{ub}) c_{ij} d_{j} y_{ijl} \leq \\ &&\sum\limits_{k \in K} (24\! \cdot\! k \sqrt[k]{q^{ub}} - 24 \!\cdot \!(k - 1) \sqrt[\!(k-1)]{q^{ub}}) x_{ik}  \forall i \in I ~~~~~~~~~\quad\quad\quad\quad~~\text{(8)}\\ &&x_{ik}, y_{ijl} \in \{0, 1\} \forall i \in I, j \in J, k \in K, l \in L \end{array} $$

The *CMEXCLP* (9) maximizes the expected number of calls reached within the time threshold across all demand nodes. The consideration of probabilistic coverage by multiplying *p*_*ij*_ represents an extension of the *MEXCLP*. Constraints (10) ensure that a number of *V* servers is distributed among all facilities. Constraints (11) state that, except for the first server, a site may only have an additional server *k* if it has server *k* − 1. Constraints (12) require that every demand node is covered on each level. A site *i* can only provide coverage to a demand node *j* if it is activated, i. e. *x*_*i*1_ = 1 (Constraints (13)). Additionally, each site *i* can provide coverage to demand node *j* only on a single level *l* (Constraints (14)). Finally, to model site interdependencies and to ensure a maximum busy fraction *q*^*ub*^, Constraints (8) is adopted from Section 3.1 according to the previous discussion. For those constraints *i* in (8) which are met with equality, the site busy probability is equal to *q*^*ub*^. The busy probability of a site decreases with an increasing slack for Constraints (8). In the objective function, we estimate the coverage using busy probability *q*^*ub*^. Since the busy probability of each site is at most *q*^*ub*^ according to Constraints (8), the objective is a worst case estimation of the expected coverage as the objective value tends to increase for lower busy probabilities. Constraints (15) limit binary variables *x*_*ik*_ and *y*_*ijl*_ to their domains. Probabilistic coverage has been proposed as an alternative to the binary definition of coverage (see [33, 35]). Binary coverage definitions assume that there is a set of demand nodes for each site that can be reached within the time threshold if a server is available. In contrast, with probabilistic coverage a probability *p*_*ij*_ is specified, which denotes that a server dispatched from site *i* will reach a call from demand node *j* within the time threshold. In order to evaluate the impact of probabilistic coverage and site interdependencies separately, we further propose the Level Maximum Expected Covering Location Problem (*LMEXCLP*). The *LMEXCLP* represents an extension of the *MEXCLP* by probabilistic coverage only. We use the *LMEXCLP* as a benchmark for the *CMEXCLP* in our computational experiments and case study. We state the mathematical formulation in the Appendix.

### A note on a chance constrained extension

In the *CMEXCLP*, the objective is to maximize the expected number of calls reached within the time threshold. We consider this objective throughout our computational experiments and case study. Nevertheless, we would like to formulate a chance constraint that is a possible extension of the *CMEXCLP*. Let *α*_*j*_ be the minimum probability that a call at demand node *j* is reached within the time threshold. Then we can state the maximum tolerable failure probability as 1 − *α*_*j*_. The following non-linear constraint limits the failure probability for each demand node *j*:

16 $$ \begin{array}{@{}rcl@{}} \prod\limits_{i \in I} \prod\limits_{l \in L} (1 - (1 - q^{ub}) p_{ij})^{y_{ijl}} \leq 1 - \alpha_{j} \quad \forall j \in J \end{array} $$

The product of the probabilities that each of the assigned sites does not reach the demand node in time must be lower or equal to the tolerable failure probability. Constraints (16) ensure that every demand node is covered at least with the probability *α*_*j*_. Similar to our capacity constraint, we rely on a uniform upper bound for the site busy probability and integrate probabilistic coverage. We may linearize the constraint in a similar manner to the chance constraint proposed in *MALP II* by ReVelle/ Hogan [4] (this version of MALP considers a locational adjustment of the busy fraction) to obtain an equivalent linear form:

17 $$ \begin{array}{@{}rcl@{}} \sum\limits_{i \in I} \sum\limits_{l \in L} \log (1 - (1 - q^{ub}) p_{ij}) y_{ijl} \leq \log (1 - \alpha_{j}) \\ \forall j \in J \end{array} $$

We can add Constraints Eq. 17 to the *CMEXCLP* to specify a maximum fraction of calls that is not reached within the time threshold for each demand node. Multiplying the right-hand side by a binary variable, the constraint can be relaxed and violations may be penalized in the objective if it is not possible to find a solution that satisfies the constraints for all demand nodes.

## Computational experiments

To evaluate our model, this section presents extensive computational experiments. We compare the performance of the *MEXCLP*, *LMEXCLP*, and *CMEXCLP* across a large set of test instances, which have been obtained by systematically varying the number of sites and ambulances. We have also evaluated other models (e.g., the models presented in [7, 8]) as a possible benchmark. A comparison with our results is difficult due to divergent model formulations, methodologies, and the scope of application. In particular, [7] use hypercube queuing models for district design and [8] focus on rural areas. We do not compare our model to [7] as they use an iterative procedure including the hypercube queuing model. We provide computational results of the *CMEXCLP* against an adjusted version of the model of [8] in Appendix A. For our urban planning area, the *CMEXCLP* greatly outperforms the model of [8].

### Data description

We use anonymized real-world data of an EMS provider for our computational experiments and case study in the next section. We subdivide the city area into |*J*| = 163 planning squares, each of which is one square kilometer in size. Among these, there are a number of |*I*| = 50 planning squares, where sites exist or additional sites could be established. Currently, an average of 13 ambulances are on duty, allocated to six ambulance stations. We consider one year of call data as the basis for our experiments. Over the period of a year, there were a total number of about 27,000 time critical emergency calls or on average approximately 3.1 calls per hour. As a model input, the Euclidean distance between planning squares only provides a rough approximation of the real street network distances. Thus, we use the *Open Street Map Routing Machine* and *Nominatim* to estimate the actual street distances between each two planning squares [44]. In order to determine the average service duration of an ambulance for an emergency, we add the pre-travel delay until the ambulance leaves the site and the time the ambulance is busy after reaching the emergency (e.g., treatment, travel time to hospital, return to site, disinfection) to the corresponding travel time. We are able to determine these model input values based on the empirical call data. For the pre-travel delay we assume a fixed time of 60 seconds (s) for each call. For the travel time we obtain an average of 425s with a standard deviation of 217s. Finally, for the average service duration we obtain a value of 3925s and a standard deviation of 1560s.

The busy fraction for the *MEXCLP* and *LMEXCLP* are computed on the basis of the historical average call duration, the historical average number of calls per day and the number of available ambulances using Equation (5). In the case of the *CMEXCLP*, the busy fraction represents a uniform upper bound for site unavailability. In order to determine the best upper bound on the site busy fraction for the *CMEXCLP*, we solve each test instance for a fixed set of parameter values. We evaluate each value for *q*^*ub*^ based on the coverage resulting from our Discrete Event Simulation (DES) as described in Section 4.2, which we will refer to as *simulated coverage* in the following. The highest simulated coverage value then defines the best *q*^*ub*^ value for each test instance.

### Discrete Event Simulation

To evaluate the quality of the solutions obtained by the different mathematical formulations for our test instances, we use a Discrete Event Simulation (DES). DES is a common methodology for analyzing the quality of model solutions in the context of EMS [45]. An emergency call is characterized mainly by the associated demand node, the position of the associated ambulance and the call duration. The dispatch of an ambulance follows the closest-idle strategy. Thus, the time that an ambulance takes to reach the scene depends directly on the location of the closest idle site. In order to simulate call arrival times, we use historical call arrival times in a trace-driven simulation approach.

Furthermore, we simulate uncertainties associated with dynamic pre-travel delays and travel times of the ambulances using distribution functions. There are many different approaches in the literature to account for randomness of travel times (see e.g., [46–48]). We use a transformation of the normal distribution for pre-travel delays and a generalized logistic distribution for travel times to account for different driving speeds of an ambulance. These distributions provide the best fit for our empirical data which has been determined using the Python statistics package *scipy* based on the minimum sum of squared errors. To estimate the travel times, the actual travel time as predicted by the Geographic Information System (GIS) software is multiplied by a time dependent travel time correction factor. These time dependent correction factors are the ratio of the historical and the GIS estimated times required for each trip. We then fitted time dependent generalized logistic distribution functions for each hour of the day based on the computed correction factors of all historical trips. On average our fitted distribution function generates a value of 0.9570 with a standard deviation of 0.3707 for the correction factor. This implies that an ambulance on average only needs 95.70% of the travel time that GIS software estimates. However, the high standard deviation implies that the actual travel times highly depend on various known and unknown factors as, e.g., traffic, weather conditions, types of road, or time of day [46, 48]. Thus, we are able to integrate empirical data and real variations in travel time in our simulation.

In order to determine the time required to reach the scene of an accident, we add the pre-travel delay to the travel time. Each simulation run then determines the response time for all historical calls over the period of one year. The total call duration, that determines when the ambulance is available after answering a call, is calculated by adding up the stochastic response time and the historical average duration it takes for an ambulance to return to its site. If the rare event occurs that a call cannot be answered by any local ambulance, it is outsourced to another EMS provider and, thus, leaves the considered system. It is a common policy between the municipalities to answer emergency calls of neighboring EMS providers when no local ambulance is available. For detailed information on the frequency of calls arriving when all local ambulances are busy, see Appendix A.

Finally, for a validation of our DES model we compared the historical coverage values with the simulated coverage as obtained by the simulation model for the actual choice of facility and vehicle locations. We compared the coverage values of more than 100 planning squares with at least 30 calls over the period of one year. On average, the simulation would indicate whether a call was covered on time incorrectly for less than 2 emergency calls per planning square per year. Thus, our simulation model provides a suitable representation of the real world EMS system.

### Results

All computational experiments were performed on a computer with an Intel Core i7-8550 with 8GB of RAM. The models were implemented in Python 3.7 and solved with Gurobi 8.1. Test instances were obtained by varying the number of sites from 4 to 12 and the number of ambulances from 6 to 12 both in single steps. Note that cases with fewer ambulances than sites are not of interest and have been neglected (for more details see Table 2). In order to determine the upper bound for the capacity constraints of the *CMEXCLP*, busy fractions from 0.05 to 0.525 were assessed in 0.025 increments. Preliminary experiments have indicated that a more detailed subdivision does not improve coverage results. Furthermore, busy fractions > 0.525 did not improve the solution quality either, as the capacity constraints are non-binding for large values of *q*^*ub*^. In order to determine the best upper bound for the site busy fraction, the model solution was evaluated by the DES for each *q*^*ub*^ value. The highest coverage value calculated by the simulation then defines the best *q*^*ub*^ value for each instance. As a result, the *MEXCLP*, *LMEXCLP*, and *CMEXCLP* were solved for a total of 42 test instances to evaluate the model performance. Note that the *CMEXCLP* model had to be solved multiple times per instance. The time limit for all computations was set to 1800 seconds.

#### Runtime and Gap

To evaluate the solution characteristics of the three mathematical formulations *MEXCLP*, *LMEXCLP*, and *CMEXCLP*, we compare their runtimes and optimality gaps. We show the results in Table 1. On average across all instances, the basic model *MEXCLP* has the shortest runtime with about 0.16 seconds, followed by *LMEXCLP* with 1157.93 seconds and *CMEXCLP* with 5577.71 seconds. The run time of the *CMEXCLP* includes all 20 sub-instances for selecting the best *q*^*ub*^ value. Thus, it takes around 280 seconds on average (= 5577.71/20) to solve one instance for a specific *q*^*ub*^ value for the *CMEXCLP* model. The time the DES takes to determine the simulated coverage in case of the *CMEXCLP* is not included, as it is applied after solving all model instances and only requires a few seconds per instance. Interestingly, the runtime of the level based formulation of *LMEXCLP* is reduced on average by adding capacity constraints given a predetermined busy fraction. The longer runtime of the *CMEXCLP* stems primarily from the iterative approach for determining the best *q*^*ub*^ value. However, the runtime strongly depends on the respective scenario. Instances with 6 to 8 possible sites are more difficult to solve because there tend to be more combinations for assigning ambulances and demand nodes to sites. Considering the *CMEXCLP* formulation, the runtime additionally depends on the selected value for *q*^*ub*^. A high value for *q*^*ub*^ means that the capacity of each site increases, as ambulances are allowed to be called more frequently. Thus, the coverage constraints can be met more easily and the instances are typically easier to solve.

**Table 1:** Overview of computational characteristics for the different mathematical formulations

	Mean	Mean	# instances
	runtime (s)	gap (%)	with gap ≥ 1%
MEXCLP	0.16	0%	0
LMEXCLP	1157.93	0.85%	12
CMEXCLP	5577.71	0.28%	40

It turns out that some instances of *LMEXCLP* could not be solved to optimality within the time limit, while the average gap is 0.85%. However, most instances of *CMEXCLP* were solved to optimality and the average gap is 0.28% across all 840 sub-instances (= 20 ∗ 42) for determining the best *q*^*ub*^ value for each instance. As a result, the test instances were solved to a reasonable degree of optimality from a practical perspective. The complexity of the *LMEXCLP* and *CMEXCLP* as compared to the *MEXCLP* stems from the combinatorial nature of the multi-level assignments. The *MEXCLP* could be solved to optimality in all instances.

#### Coverage

To assess the solution quality, we compare the level of coverage for the optimal solutions of each of the three mathematical formulations. Therefore, we use the previously described DES to determine the coverage of the network structure prescribed by the different mathematical formulations for each test instance. In our experiments, an emergency call is considered covered if it is reached within a time threshold of 10 minutes, e.g., a coverage of 90% means that 90% of all emergency calls were reached within 10 minutes. The resulting coverage for each instance is calculated as the mean of 30 simulation runs and is shown in Table 2. Table 6 in the Appendix shows that 39 out of 42 coverage improvements are significant with a 99% confidence level. The coverage results in Table 2 show that an extension of the *MEXCLP* by probabilistic coverage improves the coverage by 1.46%-points (%p) on average, while the maximum improvement amounts to 3.01%p. Adding capacity constraints improves the simulated coverage by a further 0.88%p on average and up to 2.54%p at maximum as reported in Table 2.

**Table 2:** Comparison of the simulation results for the solutions of the different mathematical formulations

				Coverage^*a*^			Δ	
S^*b*^	V^*b*^	q^*c*^	*q*^*ub*^ ^*c*^	MEXCLP	LMEXCLP	CMEXCLP	LMEXCLP -	CMEXCLP -
							MEXCLP	LMEXCLP
4	6	56.34%	47.50%	66.98%	66.71%	**68.66%**	-0.27%	1.95%
4	7	48.29%	30.00%	72.28%	72.16%	**74.70%**	-0.12%	**2.54%**
4	8	42.26%	22.50%	76.79%	78.21%	**79.89%**	1.42%	1.68%
4	9	37.56%	15.00%	81.41%	83.56%	**84.17%**	2.14%	0.61%
4	10	33.80%	20.00%	83.28%	86.10%	**86.27%**	2.82%	0.17%
4	11	30.73%	10.00%	86.10%	86.81%	**88.06%**	0.71%	1.25%
4	12	28.17%	10.00%	88.63%	88.37%	**89.05%**	-0.26%	0.68%
5	6	56.34%	50.00%	67.85%	66.93%	**68.97%**	-0.91%	2.04%
5	7	48.29%	42.50%	73.94%	74.81%	**75.74%**	0.87%	0.93%
5	8	42.26%	35.00%	78.91%	79.32%	**81.14%**	0.41%	1.83%
5	9	37.56%	27.50%	82.19%	83.22%	**84.60%**	1.04%	1.38%
5	10	33.80%	12.50%	84.46%	87.17%	**87.58%**	2.71%	0.40%
5	11	30.73%	17.50%	87.65%	88.81%	**89.97%**	1.15%	1.16%
5	12	28.17%	17.50%	90.12%	90.92%	**91.56%**	0.80%	0.64%
6	6	56.34%	50.00%	68.47%	68.98%	**69.36%**	0.51%	0.39%
6	7	48.29%	55.00%	74.50%	75.47%	**76.47%**	0.97%	1.00%
6	8	42.26%	42.50%	79.64%	80.15%	**81.97%**	0.51%	1.82%
6	9	37.56%	27.50%	82.74%	84.47%	**85.77%**	1.73%	1.31%
6	10	33.80%	40.00%	86.19%	87.90%	**88.66%**	1.71%	0.75%
6	11	30.73%	25.00%	89.21%	90.40%	**90.97%**	1.19%	0.57%
6	12	28.17%	12.50%	90.65%	**92.71%**	**92.71%**	2.06%	0.00%
7	7	48.29%	55.00%	74.51%	76.06%	**76.83%**	1.54%	0.78%
7	8	42.26%	50.00%	80.18%	80.41%	**82.45%**	0.24%	2.04%
7	9	37.56%	52.50%	83.89%	85.11%	**86.32%**	1.22%	1.21%
7	10	33.80%	27.50%	86.67%	88.24%	**89.39%**	1.57%	1.16%
7	11	30.73%	25.00%	88.79%	90.90%	**91.37%**	2.12%	0.46%
7	12	28.17%	25.00%	89.95%	92.84%	**93.13%**	2.90%	0.28%
8	8	42.26%	45.00%	80.13%	81.15%	**82.55%**	1.02%	1.40%
8	9	37.56%	50.00%	84.13%	85.39%	**87.03%**	1.26%	1.64%
8	10	33.80%	45.00%	87.38%	88.54%	**89.68%**	1.16%	1.14%
8	11	30.73%	27.50%	89.54%	91.29%	**91.83%**	1.76%	0.53%
8	12	28.17%	17.50%	90.72%	**93.26%**	**93.26%**	2.54%	0.00%
9	9	37.56%	35.00%	83.56%	86.57%	**86.72%**	**3.01%**	0.16%
9	10	33.80%	45.00%	87.11%	89.50%	**89.86%**	2.39%	0.36%
9	11	30.73%	27.50%	89.66%	91.86%	**92.06%**	2.19%	0.20%
9	12	28.17%	22.50%	91.09%	93.41%	**93.67%**	2.32%	0.26%
10	10	33.80%	32.50%	87.71%	89.40%	**90.05%**	1.69%	0.65%
10	11	30.73%	32.50%	90.16%	91.80%	**92.33%**	1.64%	0.53%
10	12	28.17%	27.50%	91.02%	93.69%	**93.83%**	2.67%	0.14%
11	11	30.73%	30.00%	89.74%	**92.52%**	**92.52%**	2.77%	0.00%
11	12	28.17%	35.00%	92.12%	93.58%	**94.09%**	1.47%	0.50%
12	12	28.17%	32.50%	91.15%	93.79%	**94.17%**	2.64%	0.38%
						average	1.46%	0.88%

^a^ Fraction of calls reached within the time threshold of 10 min

^b^ S = no. of sites, V = no. of ambulances

^c^ q = average system-wide busy fraction, *q*^*ub*^ = best upper bound for site busy fraction

**Fig. 2:**
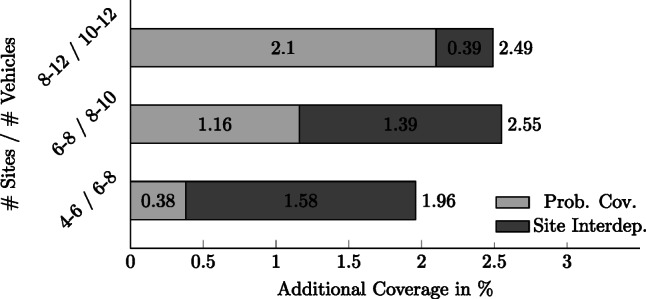
Coverage effect in %-points by model extensions of probabilistic coverage and additionally site interdependencies at different levels of resource availability

Figure 2 provides a concise illustration of some of the results from Table 2. An interesting result with respect to Fig. 2 is that capacity constraints often lead to greater improvements when resources are limited. Comparing the *CMEXCLP* with the *LMEXCLP* formulation, instances with fewer resources, i.e., 4 to 6 sites and 6 to 8 ambulances, improve coverage by 1.58%p on average, which is 0.70%p above the overall average. By contrast, in cases with many resources, i.e., 8 to 12 sites and 10 to 12 ambulances, the coverage is improved only by 0.39%p on average, which is 0.49%p below the overall average. Conversely, an extension of the *MEXCLP* by probabilistic coverage leads to a higher improvement if more resources are available. Comparing the *LMEXCLP* with the *MEXCLP*, an improvement in coverage by 0.38%p is obtained on average for instances with few resources. For instances with many resources, however, the coverage is improved by 2.10%p on average, which is highly above the average improvement of 1.46%p.

Intuitively, one of the main reasons that the improvement of the *CMEXCLP* is higher in case of limited resources is that the capacity constraints are more frequently binding. With a higher number of ambulances and sites, the capacity constraints are binding less frequently and thus have a lower impact on the solution. The consideration of probabilistic coverage is particularly important when a high number of ambulances is available. This is mainly caused by uncertain travel times leading to a lower coverage than expected by the model. When more ambulances are available, it is possible to counteract the effect of uncertain travel times by locating ambulances out over the urban area. This option is neglected by the *MEXCLP* because of its binary definition of coverage, whereas coverage is improved using the *LMEXCLP* by considering a more accurate representation of travel times. In 3 of 42 cases, the *LMEXCLP* finds the same solution as the *CMEXCLP* because the capacity constraints are non-binding for the optimal solution of the *LMEXCLP*. In these cases, there are neither advantages nor disadvantages in introducing the capacity constraints. As a result, our experiment has shown (Table 2) that the introduction of capacity constraints improves the solution in most cases and never causes a disadvantage.

Overall, the results of our computational experiments indicate that the solution quality is greatly improved by an extension of EMS location models by probabilistic coverage and capacity constraints. Most importantly, the *CMEXCLP* formulation integrates site interdependencies leading to a more accurate modeling approach. Furthermore, the solution of the new mathematical formulation is always at least as good and in most cases better than the solutions of the *LMEXCLP* and *MEXCLP*. To get further insights into the applicability of this modeling approach, the next section discusses the effects of capacity constraints and probabilistic coverage in the context of a real EMS provider.

## Case Study

**Fig. 3:**
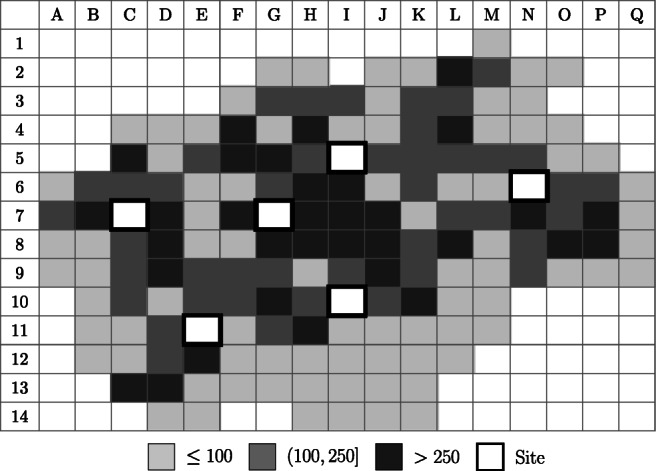
Demand distribution over the entire city and existing emergency site locations (*S*_*base*_)

To get further insights into the applicability of the new model formulation, we investigate five scenarios based on an existing EMS network. Figure 3 provides an overview of the city structure and shows the 6 existing base locations, indicated by the planning squares framed in black. In the base scenario *S*_*base*_, there are 6 existing sites and 13 ambulances. The number and color of each square indicates the number of emergency calls that occurred at that demand point over a year. As can be seen, the majority of emergencies arises near the city center while fewer calls originate in the suburbs.

### Scenario description

In this case study, we consider different scenarios to adapt the existing EMS site locations. Our analysis focuses especially on the impact of considering site interdependencies on a real EMS network. Therefore, we investigate different scenarios with a fixed number of ambulances and analyze the effects of relocating sites and constructing new sites. In all scenarios, 13 ambulances are available. To evaluate the impact of different policies, each scenario considers a different number of site constructions and site relocations. Table 3 provides an overview of the scenarios. The scenario subscript indicates the number of additional sites and relocations.

**Table 3:** Overview of case study scenarios (scenario subscript indicates number of additional sites and relocations)

	Scenario				
	*S*_*base*_	*S*_0/1_	*S*_0/2_	*S*_1/0_	*S*_1/1_
Existing sites	6	6	6	6	6
Additional sites	0	0	0	1	1
Relocations	0	1	2	0	1

The base scenario *S*_*base*_ represents the base case with six existing sites. In scenarios *S*_0/1_ and *S*_0/2_, respectively, one or two relocations of existing sites are permitted. In total, the number of sites in these cases remains at six. Scenario *S*_1/0_ allows for an additional site while the existing ones are retained. Finally, scenario *S*_1/1_ allows for one relocation and the construction of one new site. Thus, scenarios *S*_1/0_ and *S*_1/1_ result in a total number of seven sites. Note that in all scenarios no more than two new sites are established, either by moving existing sites, or by constructing new ones, or a combination of both. From a practical point of view, scenarios that require the construction of more than two new sites are not realistic, as these scenarios would require excessive investment and impose a large change on the existing system. In the base case, the locations are predetermined.

The optimal ambulance allocation has to be chosen, which may differ from the

existing ambulance allocation. In all other scenarios, at least one additional site may be established.

### Results and implications

In the following, we evaluate the model solutions of the five scenarios described above. We solve each of the three models for each scenario and evaluate the solution using the DES described in Section 4.

Table 4 shows the solutions of the different model formulations. The values represent the number of ambulances located at the site indicated by the column value in the respective scenario that is given by the row of each model formulation block. In the majority of cases where site relocations are permitted, the same locations are selected by the different model formulations. On the other hand, the ambulance allocation is different for almost all the scenarios. We use these site locations and ambulance allocations as input for the DES to evaluate the performance of each emergency network.

**Table 4:** Optimal site locations and ambulance allocations of the *MEXCLP*, *LMEXCLP*, and *CMEXCLP* for each scenario

		Number of ambulances at Planning square (see Fig. 3)										
		C7	C10	C12	D8	E11	G7	G10	I5	I10	K5	N6
MEXCLP	*S*_*base*_				2		1	3	3	1		3
	*S*_0/1_		2		2			1	2	2		3
	*S*_0/2_		2		2			2		1	3	3
	*S*_1/0_		2		2		1	1	2	2		3
	*S*_1/1_		2		2			2	1	1	2	3
LMEXCLP	*S*_*base*_				2		2	2	2	2		3
	*S*_0/1_	3					1	2	2	2		3
	*S*_0/2_	2				1	3		2	2		3
	*S*_1/0_	2			1		1	2	2	2		3
	*S*_1/1_	2		2			1	1	2	2		3
CMEXCLP	*S*_*base*_				2		2	2	3	2		2
	*S*_0/1_	2					2	2	3	2		2
	*S*_0/2_	2				2	2		3	2		2
	*S*_1/0_	2			1		2	2	2	2		2
	*S*_1/1_	2				2	2	1	2	2		2

^a^ PS = Planning square

Table 5 shows the difference of the coverage in the simulation between the models for the different scenarios. The extension of *MEXCLP* to *LMEXCLP* by probabilistic coverage improves the resulting coverage by 2.31%p on average. Scenario *S*_0/2_ obtains the highest improvement of 4.78%p coverage in comparison to the *MEXCLP* model. In case of site interdependencies, we obtain an additional improvement. The *CMEXCLP* considers both probabilistic coverage and interdependencies between emergency sites and improves the coverage by a further 0.59%p on average compared to the *LMEXCLP*. Site interdependencies lead to the highest improvement in scenario *S*_0/2_, which allows for the relocation of two existing sites. In this instance, the simulated coverage site increases by 1.05%p compared to the *LMEXCLP*. If all ambulances of a certain site are busy, the probability that a nearby site will have to deploy an ambulance to answer a call increases. Limiting the site busyness and thereby providing enough ambulances to answer primary as well as additional calls from other sites leads to a further improvement of the coverage provided by the network.

**Table 5:** Comparison of the additional effects on the coverage obtained by the different mathematical formulations in the case study scenarios

	*Δ*		
	LMEXCLP -	CMEXCLP -	CMEXCLP -
	MEXCLP	LMEXCLP	MEXCLP
*S*_*base*_	0.35%	0.45%	0.80%
*S*_0/1_	1.81%	0.54%	2.35%
*S*_0/2_	**4.78%**	**1.05%**	**5.83%**
*S*_1/0_	1.36%	0.16%	1.52%
*S*_1/1_	3.24%	0.74%	3.98%
average	2.31%	0.59%	2.89%

**Fig. 4:**
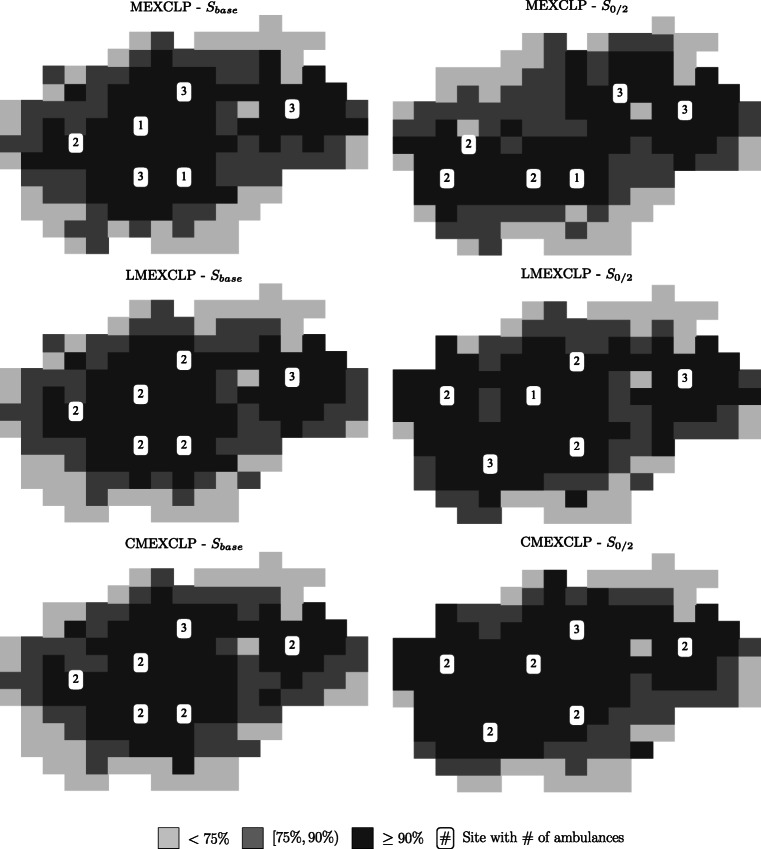
Comparison of location and allocation decisions with resulting levels of coverage provided by *MEXCLP*, *LMEXCLP*, and *CMEXCLP* model solutions in scenarios *S*_*base*_ and *S*_0/2_

To further illustrate the effects of explicitly modeling probabilistic coverage and site interdependencies, we compare the optimal ambulance allocation prescribed by the mathematical models (*CMEXCLP*, *LMEXCLP*, and *MEXCLP*) and the resulting simulated coverage for each planning square in Fig. 4 for the two scenarios *S*_*base*_ and *S*_0/2_.

This figure illustrates the city structure with the color of each planning square representing the simulated coverage obtained by the corresponding model solutions and the selected site locations as white boxes. We represent the number of allocated ambulances by the value inside these boxes. Note that these planning squares are covered ≥ 90% as well.

An interesting observation resulting from this figure is that the explicit consideration of site interdependencies often leads to the same site locations compared to the *LMEXCLP* but with a more efficient allocation of ambulances. Especially in scenario *S*_0/2_, the *MEXCLP* reduces the available resources near the city center and moves sites and ambulances to planning squares further away from the center. Taking into account variations of the travel speed, by introducing probabilistic coverage, the *LMEXCLP* locates more ambulances near the city center compared to the *MEXCLP* formulation. Even in the base scenario *S*_*base*_, the simulated coverage increases by 0.45%p (Table 5), if site interdependencies are explicitly taken into account. This improvement is exclusively obtained by an enhanced ambulance allocation to existing emergency sites. The main reason for that is that in the base scenario (*S*_*base*_) most sites are located near the city center. Due to the assumption of a system-wide busy fraction, the *MEXCLP* and *LMEXCLP* overestimate the coverage of the demand in the city center. Thus, only few ambulances are located at existing sites near the city center while most are allocated in the suburbs. As a result, the *CMEXCLP* formulation improves the coverage.

**Fig. 5:**
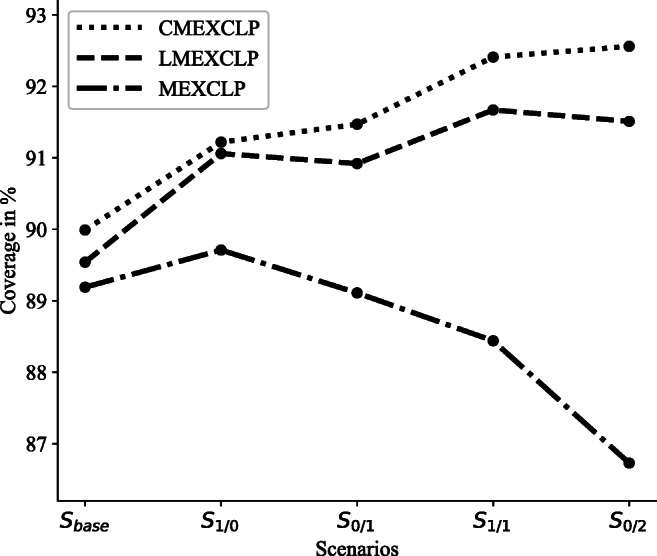
Coverage provided by the model solutions for each scenario

Looking at Fig. 5, it is interesting to note that the relocation (*S*_0/1_) or construction of one site (*S*_1/0_) and the relocation of two sites (*S*_0/2_) or construction and relocation of each one site (*S*_1/1_) lead to very similar simulated coverage results for the *LMEXCLP* and *CMEXCLP* formulations. It seems that the positive effect of an additional site is limited by the given number of ambulances. Furthermore, the main benefit appears to result from adding new sites to the network, while some existing sites can be dropped without a large impact on the coverage provided by the network. In the considered scenarios, it is more beneficial to maintain a number of six sites by relocating one or two sites than to construct additional sites. Thus, from a practical point of view, decisions on resources should consider both the number of sites and ambulances. One result that is somewhat counterintuitive is the deterioration in coverage of the *MEXCLP* in scenarios *S*_0/1_, *S*_0/2_, and *S*_1/1_. It is likely that this reduction is due to the error inherent in the assumption of a system-wide busy fraction combined with a binary coverage formulation. This further illustrates the importance of explicit consideration of probabilistic coverage and site interdependencies in modeling EMS networks.

Overall, both extensions of the *MEXCLP* model have a significant influence on the network quality. The results of this study show that the explicit consideration of site interdependencies is highly relevant in real EMS systems and leads to improved location solutions, especially in case of limited resources. The *CMEXCLP* model provides the highest value for simulated coverage in all considered scenarios and is therefore an important tool for planning EMS networks. Further, the *CMEXCLP* determines a solution that achieves a higher coverage with an identical number of ambulances than the solution of *MEXCLP* or *LMEXCLP*, i.e., it attains better performance at the same cost.

## Conclusion and Outlook

The new mathematical formulation *CMEXCLP* explicitly models site interdependencies by introducing new upper bound chance constraints on the busyness of each site. Furthermore, we model probabilistic coverage leading to a more realistic representation of the EMS system by the mathematical formulations.

In extensive computational experiments, we have separately evaluated the effect of modeling probabilistic coverage and site interdependencies. Probabilistic coverage has often improved the solution. However, we have obtained the best solution for each test instance by taking into account site interdependencies. While the size of the effect varies, the solution has been improved in comparison to the other mathematical formulations. The new mathematical model leads to large enhancements of coverage, in particular when there are strongly limited resources. Especially, the assignment of ambulances to sites is often superior to previous formulations due to the more accurate representation of capacities. As a result, site interdependencies are an important feature when optimizing an EMS system with a heterogeneous demand distribution, since their consideration may lead to a substantial improvement of coverage.

In our case study, we have demonstrated the value of the new mathematical formulation for a real EMS system. It turns out that two relocations lead to the largest improvement over the base case.

Future research should look into further extensions of the *CMEXCLP* like for example time dependencies. Moreover, the value of the proposed formulation should be investigated by applications to more EMS systems. Futhermore, the possibility of overfitting could be analyzed by taking into account training and testing samples to separate the DES input estimation from the DES model selection that determines the best solution. Finally, specialized solution approaches are required for large instances, especially when chance constraints on the minimum fraction of coverage at each demand node are introduced.

## Appendix

### Symbol directory

**Table:**

Name	Description
*I*	Set of potential sites
*J*	Set of demand nodes
*K*	Set of capacity levels at each site
*L*	Set of coverage levels
*α*	Probability that a call is served
	within the required time
*c*_*i**j*_	Average total time required to
	answer a call at demand node *j* from site *i*
*d*_*j*_	Number of calls at node *j*
*f*_*i**j*_	Number of calls at demand node *j*
	covered by site *i* per day
*N*_*j*_	Set of sites *i* covering demand node *j*
*p*_*i**j*_	Probability that a server from site *i*
	answers a call at demand node *j* in time
*q*	Busy probability
*q*^*u**b*^	Upper bound for the busy fraction
$\bar {t}$	Average duration per call (in hours)
*V*	Total number of servers that are available
$x_{i} \in {\mathbb {Z}}^{\geq 0}$	Integer variable that denotes the
	number of servers at site *i*
*x*_*i**k*_ ∈{0, 1}	1 if server number *k* is added at site
	*i*; 0 otherwise
*y*_*j**k*_ ∈{0, 1}	1 if demand node *j* is covered by *k*
	servers; 0 otherwise
*y*_*i**j**l*_ ∈{0, 1}	1 if site *i* serves demand node *j* on
	level *l*; 0 otherwise
*y*_*i**k**j**l*_ ∈{0, 1}	1 if site *i* serves demand node *j* with
	a number of *k* ambulances on level
	*l*; 0 otherwise
*z*_*j*_ ∈{0, 1}	1 if calls at demand node *j* are reached with a reliability of at least *α*; 0 otherwise

### LMEXCLP

The *LMEXCLP* is an extension of the *MEXCLP* by probabilistic coverage. In contrast to the *CMEXCLP*, an additional index is required for variable *y*_*i**k**j**l*_. Since there is no system-wide site busy fraction, the number of servers that provide coverage from site *i* for demand point *j* on level *l* needs to be captured by index *k*. Then, the coverage provided by site *i* takes into account the number of servers. In that way, the additional effect of locating multiple servers at a single site can be represented.

18 $$ \begin{array}{@{}rcl@{}} &\max\quad & \sum\limits_{i \in I} \sum\limits_{k \in K} \sum\limits_{j \in J} \sum\limits_{l \in L} (1 - q) q^{l} d_{j} p_{ij} y_{ikjl} \end{array} $$

19 $$ \begin{array}{@{}rcl@{}} &s. t. & \sum\limits_{i \in I} \sum\limits_{k \in K} x_{ik} = V \end{array} $$

20 $$ \begin{array}{@{}rcl@{}} &&x_{ik} \leq x_{i(k-1)} \quad \forall i \in I, k \in K\ |\ k > 1 \end{array} $$

21 $$ \begin{array}{@{}rcl@{}} && \sum\limits_{i \in I} \sum\limits_{k \in K} y_{ikjl} = 1 \quad \forall j \in J, l \in L \end{array} $$

22 $$ \begin{array}{@{}rcl@{}} && y_{ikjl} \leq x_{ik} \quad \forall i \in I, j \in J, k \in K, l \in L \end{array} $$

23 $$ \begin{array}{@{}rcl@{}} && \sum\limits_{l \in L} y_{ikjl} \leq 1 \quad \forall i \in I, j \in J, k \in K \end{array} $$

24 $$ \begin{array}{@{}rcl@{}} &&x_{ik}, y_{ikjl} \in \{0, 1\} \\ && \qquad\qquad\quad\qquad\forall i \in I, j \in J, k \in K, l \in L \end{array} $$

### Confidence intervals

Table 6 shows the confidence intervals of our computational experiments. Since the coverage results are simulated, we test the improvement of the CMEXCLP model compared to the MEXCLP and LMEXCLP for statistical significance. Therefore, we conduct two t-tests with a confidence level of 99.5% to test the significance of the improvement of the CMEXCLP in comparison to the MEXCLP and LMEXCLP. We apply the t-test to the difference between the individually simulated coverage results to reduce the variance. If both tests are significant it is valid to state that the CMEXCLP is significantly better at a 99% confidence level in comparison to both models. The test results show, that in 39 out of 42 instances the CMEXCLP is significantly better at a 99% confidence level compared to the MEXCLP and LMEXCLP.

**Table 6:** Results of the 99% confidence intervals calculations for the differences of the simulated coverage values of our computational experiments (Std Dev = standard deviation, t-values = values of the student t distribution with *α* = 0.005 and 30 runs, CI lb = confidence interval lower bound, CI ub = confidence interval upper bound, significant = if the 0 value is not included in the confidence interval for both comparisons at a 99,5% confidence level it follows that the average coverage value of the CMEXCLP is significantly better at a 99% confidence level compared to both LMEXCLP and MEXCLP models)

		*Δ* CMEXCLP-MEXCLP						*Δ* CMEXCLP-MEXCLP						
Sites	Vehicles	average	Std Dev	t-value	CI lb	CI ub	significant	average	Std Dev	t-value	CI lb	CI ub	significant	CMEX is significantly better on
														99% confidence level
4	6	0.0195	0.0033	0.0018	0.0177	0.0214	Yes	0.0168	0.0026	0.0015	0.0154	0.0183	Yes	Yes
4	7	0.0254	0.0033	0.0018	0.0236	0.0272	Yes	0.0242	0.0025	0.0014	0.0228	0.0256	Yes	Yes
4	8	0.0168	0.0034	0.0019	0.0149	0.0187	Yes	0.0310	0.0029	0.0016	0.0294	0.0326	Yes	Yes
4	9	0.0061	0.0025	0.0014	0.0047	0.0075	Yes	0.0276	0.0033	0.0018	0.0257	0.0294	Yes	Yes
4	10	0.0017	0.0024	0.0013	0.0004	0.0030	Yes	0.0299	0.0030	0.0017	0.0282	0.0316	Yes	Yes
4	11	0.0125	0.0028	0.0016	0.0109	0.0140	Yes	0.0196	0.0025	0.0014	0.0182	0.0210	Yes	Yes
4	12	0.0068	0.0018	0.0010	0.0058	0.0078	Yes	0.0042	0.0023	0.0013	0.0029	0.0055	Yes	Yes
5	6	0.0204	0.0028	0.0016	0.0188	0.0220	Yes	0.0113	0.0026	0.0015	0.0098	0.0127	Yes	Yes
5	7	0.0093	0.0025	0.0014	0.0079	0.0107	Yes	0.0180	0.0032	0.0018	0.0162	0.0198	Yes	Yes
5	8	0.0183	0.0034	0.0019	0.0164	0.0202	Yes	0.0224	0.0028	0.0016	0.0208	0.0240	Yes	Yes
5	9	0.0138	0.0024	0.0013	0.0125	0.0151	Yes	0.0242	0.0022	0.0012	0.0229	0.0254	Yes	Yes
5	10	0.0040	0.0018	0.0010	0.0031	0.0050	Yes	0.0312	0.0023	0.0013	0.0299	0.0324	Yes	Yes
5	11	0.0116	0.0022	0.0012	0.0104	0.0128	Yes	0.0231	0.0020	0.0011	0.0220	0.0242	Yes	Yes
5	12	0.0064	0.0020	0.0011	0.0053	0.0075	Yes	0.0144	0.0020	0.0011	0.0133	0.0155	Yes	Yes
6	6	0.0039	0.0024	0.0013	0.0025	0.0052	Yes	0.0090	0.0025	0.0014	0.0076	0.0103	Yes	Yes
6	7	0.0100	0.0025	0.0014	0.0086	0.0114	Yes	0.0197	0.0025	0.0014	0.0183	0.0211	Yes	Yes
6	8	0.0182	0.0032	0.0018	0.0164	0.0200	Yes	0.0233	0.0029	0.0016	0.0217	0.0249	Yes	Yes
6	9	0.0131	0.0023	0.0013	0.0118	0.0143	Yes	0.0304	0.0028	0.0015	0.0288	0.0319	Yes	Yes
6	10	0.0075	0.0019	0.0011	0.0065	0.0086	Yes	0.0247	0.0028	0.0015	0.0231	0.0262	Yes	Yes
6	11	0.0057	0.0022	0.0012	0.0044	0.0069	Yes	0.0175	0.0022	0.0012	0.0163	0.0187	Yes	Yes
6	12	-0.0005	0.0020	0.0011	-0.0016	0.0006	No	0.0206	0.0024	0.0013	0.0193	0.0219	Yes	No
7	7	0.0078	0.0024	0.0013	0.0064	0.0091	Yes	0.0232	0.0025	0.0014	0.0218	0.0246	Yes	Yes
7	8	0.0204	0.0025	0.0014	0.0190	0.0218	Yes	0.0227	0.0027	0.0015	0.0212	0.0243	Yes	Yes
7	9	0.0121	0.0021	0.0012	0.0110	0.0133	Yes	0.0243	0.0029	0.0016	0.0227	0.0260	Yes	Yes
7	10	0.0116	0.0021	0.0011	0.0104	0.0127	Yes	0.0273	0.0025	0.0014	0.0259	0.0287	Yes	Yes
7	11	0.0046	0.0025	0.0014	0.0032	0.0060	Yes	0.0258	0.0022	0.0012	0.0246	0.0270	Yes	Yes
7	12	0.0028	0.0016	0.0009	0.0019	0.0037	Yes	0.0318	0.0020	0.0011	0.0307	0.0329	Yes	Yes
8	8	0.0140	0.0021	0.0012	0.0129	0.0152	Yes	0.0243	0.0030	0.0017	0.0226	0.0260	Yes	Yes
8	9	0.0164	0.0028	0.0016	0.0149	0.0180	Yes	0.0290	0.0020	0.0011	0.0279	0.0301	Yes	Yes
8	10	0.0114	0.0018	0.0010	0.0103	0.0124	Yes	0.0230	0.0026	0.0014	0.0215	0.0244	Yes	Yes
8	11	0.0053	0.0017	0.0010	0.0044	0.0063	Yes	0.0229	0.0023	0.0013	0.0216	0.0242	Yes	Yes
8	12	0.0004	0.0022	0.0012	-0.0008	0.0016	No	0.0254	0.0025	0.0014	0.0240	0.0268	Yes	No
9	9	0.0016	0.0023	0.0013	0.0003	0.0028	Yes	0.0316	0.0027	0.0015	0.0301	0.0331	Yes	Yes
9	10	0.0036	0.0021	0.0012	0.0024	0.0047	Yes	0.0275	0.0020	0.0011	0.0263	0.0286	Yes	Yes
9	11	0.0020	0.0020	0.0011	0.0009	0.0031	Yes	0.0239	0.0023	0.0013	0.0227	0.0252	Yes	Yes
9	12	0.0026	0.0020	0.0011	0.0015	0.0037	Yes	0.0258	0.0021	0.0011	0.0246	0.0269	Yes	Yes
10	10	0.0065	0.0022	0.0012	0.0053	0.0077	Yes	0.0234	0.0026	0.0014	0.0220	0.0249	Yes	Yes
10	11	0.0053	0.0023	0.0013	0.0040	0.0066	Yes	0.0217	0.0026	0.0014	0.0202	0.0231	Yes	Yes
10	12	0.0014	0.0019	0.0010	0.0004	0.0025	Yes	0.0281	0.0015	0.0008	0.0273	0.0289	Yes	Yes
11	11	0.0002	0.0012	0.0007	-0.0004	0.0009	No	0.0278	0.0015	0.0009	0.0269	0.0286	Yes	No
11	12	0.0050	0.0018	0.0010	0.0040	0.0060	Yes	0.0197	0.0020	0.0011	0.0186	0.0208	Yes	Yes
12	12	0.0038	0.0020	0.0011	0.0027	0.0049	Yes	0.0302	0.0017	0.0010	0.0292	0.0311	Yes	Yes

### Dropped calls

Table 7 shows the fraction of calls that were not answered by the EMS system because all local servers were busy for each instance. Each value represents the average fraction of total calls that were dropped across 30 simulation runs per model formulation and instance.

**Table 7:** Average fraction of dropped calls that arrive when all servers in the system are busy (average of 30 simulation runs)

		average dropped calls		
Sites	Vehicles	MEXCLP	LMEXCLP	CMEXCLP
4	6	12.18%	12.19%	11.98%
4	7	7.07%	7.08%	6.96%
4	8	3.69%	3.65%	3.60%
4	9	1.80%	1.75%	1.73%
4	10	0.79%	0.76%	0.74%
4	11	0.30%	0.29%	0.28%
4	12	0.10%	0.11%	0.10%
5	6	12.12%	12.23%	12.11%
5	7	7.07%	7.03%	6.93%
5	8	3.68%	3.64%	3.61%
5	9	1.79%	1.77%	1.77%
5	10	0.78%	0.75%	0.74%
5	11	0.30%	0.28%	0.27%
5	12	0.10%	0.10%	0.09%
6	6	12.10%	12.12%	12.07%
6	7	7.04%	6.94%	6.92%
6	8	3.66%	3.61%	3.55%
6	9	1.81%	1.75%	1.71%
6	10	0.77%	0.76%	0.72%
6	11	0.28%	0.29%	0.27%
6	12	0.10%	0.09%	0.09%
7	7	7.06%	6.94%	6.89%
7	8	3.64%	3.64%	3.54%
7	9	1.78%	1.73%	1.69%
7	10	0.77%	0.75%	0.71%
7	11	0.29%	0.29%	0.27%
7	12	0.10%	0.08%	0.08%
8	8	3.65%	3.63%	3.55%
8	9	1.77%	1.74%	1.68%
8	10	0.76%	0.75%	0.71%
8	11	0.28%	0.28%	0.26%
8	12	0.09%	0.09%	0.09%
9	9	1.79%	1.73%	1.71%
9	10	0.77%	0.73%	0.72%
9	11	0.28%	0.28%	0.26%
9	12	0.09%	0.09%	0.09%
10	10	0.77%	0.74%	0.72%
10	11	0.27%	0.28%	0.27%
10	12	0.09%	0.09%	0.07%
11	11	0.29%	0.27%	0.26%
11	12	0.09%	0.09%	0.08%
12	12	0.10%	0.09%	0.08%

### Model benchmark

We adapted the original “TUBUL” model formulation of Shariat-Mohaymany et al. [8], to make their model applicable to our case and enable a comparison with CMEXCLP. In reformulating their model several changes were required; however, we aimed at replicating their basic assumptions as closely as possible. These are listed in the following. We have adopted the notation of Shariat-Mohaymany. See their paper for a definition of the basic notation [8]. First, we reformulated the objective function to increase comparability with the MEXCLP objective function (see Objective (25)). In addition, we integrated probabilistic coverage (*p*_*j**i*_) in the objective function. Further, we added constraints on the number of sites and vehicles (Equations (35) and (36)). Other constraints remain unchanged with respect to the original formulation. The mathematical formulation of Shariat-Mohaymani et al. [8] has been adjusted in the following way:

25 $$ \begin{array}{@{}rcl@{}} &\max\quad & \sum\limits_{i \in I} \sum\limits_{a \in A} d_{ia} p_{ji} \end{array} $$

26 $$ \begin{array}{@{}rcl@{}} &s. t. & \sum\limits_{a \in N{i}} x_{a} = \sum\limits_{l \in L} (l + 1) y_{il} \quad \forall i \in I \end{array} $$

27 $$ \begin{array}{@{}rcl@{}} && \sum\limits_{l \in L} y_{il} = 1 \quad\forall i \in I \end{array} $$

28 $$ \begin{array}{@{}rcl@{}} && z_{j} \geq x_{a} \quad \forall j \in J, a \in A_{j} \end{array} $$

29 $$ \begin{array}{@{}rcl@{}} && 24 p \geq \sum\limits_{i \in B{a}} d_{ia} \quad \forall a \in A \end{array} $$

30 $$ \begin{array}{@{}rcl@{}} && d_{ia} = 0 \quad \forall i \in I, a \in A \mid i \not\in B_{a} \end{array} $$

31 $$ \begin{array}{@{}rcl@{}} && d_{ia} \geq q_{i} \sum\limits_{l \in L} y_{il} \frac{1}{1+l} + (x_{a} - 1) M \end{array} $$

32 $$ \begin{array}{@{}rcl@{}} && \forall a \in A, i \in B_{a} \\ && d_{ia} \leq q_{i} \sum\limits_{l \in L} y_{il} \frac{1}{1+l} \quad \forall a \in A, i \in B_{a} \end{array} $$

33 $$ \begin{array}{@{}rcl@{}} && d_{ia} \leq M x_{a} \quad \forall i \in I, a \in A \end{array} $$

34 $$ \begin{array}{@{}rcl@{}} && d_{ia} \leq q_{i}z_{j} \quad \forall i\in I, a \in A_{j}, j \in J \end{array} $$

35 $$ \begin{array}{@{}rcl@{}} && \sum\limits_{a \in A} x_{a} = V \end{array} $$

36 $$ \begin{array}{@{}rcl@{}} && \sum\limits_{j \in J} z_{j} = S \end{array} $$

37 $$ \begin{array}{@{}rcl@{}} && x_{a}, z_{j}, y_{il} \in{\{0, 1\}} \quad \forall a \in A, i \in I, j \in J, l \in L \end{array} $$

38 $$ \begin{array}{@{}rcl@{}} && d_{ia} \geq 0 \quad \forall i \in B_{a}, a \in A \end{array} $$

We have performed the same set of computational experiments for the adapted TUBUL, using identical parameters as input and our discrete event simulation for the evaluation of the solutions. In Table 8, we provide an overview of the model performance in comparison to CMEXCLP.

**Table 8:** Comparison of simulated coverage results of CMEXCLP and adjusted TUBUL

				Coverage		*Δ*
Sites	Vehicles	*q*_*u**b*_* TUBUL	q MEX	TUBUL	CMEXCLP	TUBUL - CMEXCLP
4	6	None	56,34%	None	68,66%	None
4	7	None	48,29%	None	74,70%	None
4	8	None	42,26%	None	79,89%	None
4	9	None	37,56%	None	84,17%	None
4	10	None	33,80%	None	86,27%	None
4	11	None	30,73%	None	88,06%	None
4	12	None	28,17%	None	89,05%	None
5	6	None	56,34%	None	68,97%	None
5	7	None	48,29%	None	75,74%	None
5	8	None	42,26%	None	81,14%	None
5	9	None	37,56%	None	84,60%	None
5	10	None	33,80%	None	87,58%	None
5	11	None	30,73%	None	89,97%	None
5	12	None	28,17%	None	91,56%	None
6	6	None	56,34%	None	69,36%	None
6	7	None	48,29%	None	76,47%	None
6	8	60,00%	42,26%	72,86%	81,97%	-9,11%
6	9	55,00%	37,56%	78,02%	85,77%	-7,76%
6	10	42,50%	33,80%	82,33%	88,66%	-6,33%
6	11	40,00%	30,73%	84,88%	90,97%	-6,09%
6	12	35,00%	28,17%	87,14%	92,71%	-5,57%
7	7	65,00%	48,29%	67,47%	76,83%	-9,36%
7	8	52,50%	42,26%	75,94%	82,45%	-6,51%
7	9	47,50%	37,56%	81,52%	86,32%	-4,80%
7	10	62,50%	33,80%	85,31%	89,39%	-4,08%
7	11	35,00%	30,73%	88,40%	91,37%	-2,96%
7	12	42,50%	28,17%	89,86%	93,13%	-3,27%
8	8	50,00%	42,26%	76,56%	82,55%	-5,99%
8	9	42,50%	37,56%	82,45%	87,03%	-4,58%
8	10	40,00%	33,80%	85,77%	89,68%	-3,90%
8	11	35,00%	30,73%	89,08%	91,83%	-2,75%
8	12	35,00%	28,17%	90,86%	93,26%	-2,40%
9	9	42,50%	37,56%	82,51%	86,72%	-4,22%
9	10	37,50%	33,80%	85,49%	89,86%	-4,37%
9	11	35,00%	30,73%	89,03%	92,06%	-3,03%
9	12	40,00%	28,17%	91,45%	93,67%	-2,22%
10	10	42,50%	33,80%	86,60%	90,05%	-3,46%
10	11	65,00%	30,73%	89,40%	92,33%	-2,93%
10	12	35,00%	28,17%	91,30%	93,83%	-2,53%
11	11	35,00%	30,73%	88,95%	92,52%	-3,58%
11	12	35,00%	28,17%	91,65%	94,09%	-2,44%
12	12	47,50%	28,17%	91,57%	94,17%	-2,60%

For our case, their model does not obtain reasonable solutions. The primary reason is that they consider the assumption of evenly distributed workload of a region between all servers in proximity, combined with a set of constraints that require every planning square to be covered. They make this assumption for an area of rural outskirts with few emergencies; it turns out to be inefficient for the densely populated city in our case study. For experiments with a low number of sites or vehicles, this may even lead to an infeasible model, as there are insufficient capacities.
